# The changing epidemiology of child and adolescent mental health requires an immediate policy response

**DOI:** 10.1016/j.puhip.2025.100655

**Published:** 2025-09-25

**Authors:** Alina Cosma, Michelle Black, Stanislava Vuckovic, Ivana Pavic, Helena Fonseca, Marzia Lazzerini

**Affiliations:** a Trinity Centre for Global Health, School of Psychology, Trinity College Dublin, Dublin, Ireland; b Olomouc University Social Health Institute (OUSHI), Olomouc, Czechia; c Department of Public Health Policy and Systems, University of Liverpool, UK; d UNICEF Office in Serbia, Belgrade, Serbia; e Croatian Institute of Public Health, Zagreb, Croatia; f Department of Paediatrics, Adolescent Medicine Division, Faculty of Medicine, University of Lisbon, WHO Collaborating Centre for Adolescent Medicine and Training, Portugal; g London School of Hygiene & Tropical Medicine, London, UK; h Institute for Maternal and Child Health IRCCS “Burlo Garofolo”, WHO Collaborating Centre for Maternal and Child Health, Trieste, Italy

**Keywords:** Child and adolescent mental health, Cross-sectoral, Health policies, Europe

## Abstract

The epidemiology of child and adolescent mental health is rapidly evolving, necessitating urgent and coordinated responses across health, education, social services, and justice sectors. This commentary highlights key trends in adolescent mental health, emphasizing the shifting social, economic, and technological determinants shaping youth mental health. Recent evidence indicates a rise in mental health challenges, with increased gender disparities, heightened stress from academic pressures, social media exposure, and economic inequalities, among others, shaping adolescent mental health trajectories. Simultaneously, health systems are facing escalating demands for mental health care, compounded by workforce shortages and gaps in training to address emerging conditions such as digital addiction and societal withdrawal. The commentary underscores the importance of preventive, intersectoral, and life course approaches to mental health, advocating for increased investment in prevention, research, workforce development, and integrated care models. Addressing these issues requires evidence-based policies that consider cross-national differences and gendered trends while ensuring sustainable mental health systems for future generations. By prioritizing adolescent mental health within broader public health agendas, we can create a foundation for long-term well-being and social sustainability.

## 1 Introduction

This commentary explores the shifting epidemiology of child and adolescent mental health and offers insights into how health systems in coordination with various other sectors such as education, social services, and justice can respond effectively in addressing the European regional child and adolescent mental health needs. By addressing critical areas, such as changing determinants of adolescent mental health, rising demands on health care systems, and changes in adolescent mental health provision, we provide actionable recommendations for policymakers, and health care stakeholders to create a future where child and adolescent mental health is prioritized and protected (see Table 1).

**Table 1:** Actionable key recommendations for policymakers, and health care stakeholders.

*Why* [[1], [2], [3], [4], [5], [6], [7], [8], [9], [10], [11], [12], [13], [14], [15], [16], [17], [18], [19], [20], [21], [22], [23], [24], [25], [26]]^a^	• Mental health of young people is increasingly worsening • The determinants are many, changing, complex, and often interrelated • The national health systems are unprepared, and struggling to meet these new needs/determinants • Investment cases have showed the benefit of investing in mental health

***How***	Under the umbrella of national and international frameworks for child and adolescent (mental) health

***What*** [[27], [28], [29], [30], [31], [32], [33], [34], [35], [36], [37], [38], [39], [40], [41], [42], [43]]^a^	1 Governments must prioritize the mental health of children, adolescents, and young adults. 2 Investments are needed to support mental health services across prevention, treatment, and promotion. 3 Mental health services must be made accessible and affordable, with minimal bureaucratic barriers to care. 4 Standards of care and clear performance indicators should be adopted, aligned with international standards. 5 Well-trained, supported, and adequately resourced mental health support workforces are critical. 6 A life course intergenerational approach is needed recognising that parental mental health is a key determinant of child mental health 7 A multisectoral approach should be implemented, involving coordination across different sectors: health, education, social services, and justice. 8 Investments are needed into support systems throughout daily life, such as schools, which play a crucial role, and other systems (cultural organizations, youth groups, sports clubs, etc). 9 Mental health literacy should be promoted for reducing stigma, encouraging help-seeking behaviour, and early detection. 10 Communities should be empowered to take ownership of mental health care, promoting active involvement of children, adolescents, and their families in the development and delivery of mental health programs.

a Note: References cited in the text.

### 1.1 Changing determinants of child and adolescent mental health

**Fig. 1:**
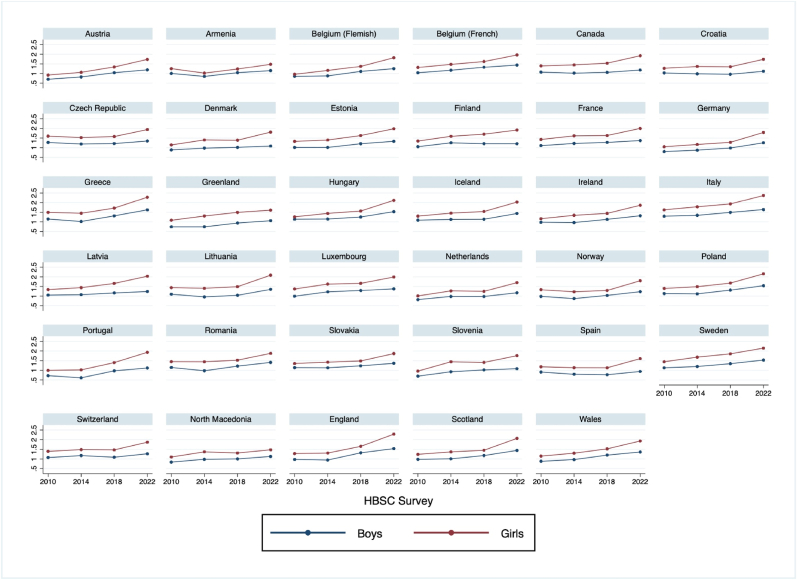
Time trends in Mean Psychological Health Complaints (2010–2022); by country and gender. HBSC data. Source https://www.sciencedirect.com/science/article/pii/S1054139X24004622.

The mental health of children and adolescents in Europe is increasingly recognized as a public health priority that is shaped by a complex and ever evolving landscape [[1], [2], [3], [4]], with a plethora of resources to support policy and service development (See Table 2). Adolescence (10–19-years-old) is a pivotal stage in the life course, marked by rapid developmental transitions and heightened vulnerability to external stressors. The mental health of adolescents is profoundly shaped by the social, economic, and commercial determinants at individual, community, national and international levels. Over the past decade, there has been a notable rise in the prevalence of mental health problems among young people, coupled with a decline in overall well-being [5] (See Fig. 1). For example, recent estimates suggested that 1 in 4 youth globally are experiencing clinically elevated depression symptoms, while 1 in 5 youth are experiencing clinically elevated anxiety symptoms [6]. Social media use, academic pressures, increased geo-political instability and polarisation, increases in the cost of living combined with inequalities within and across countries, and global challenges like climate anxiety, pandemics, war and conflicts, among others, seem to be reshaping the mental health determinants for this generation [7]. These changes call for innovative and adaptive approaches to safeguard and promote mental health in a way that reflects the realities of today's adolescents, and underline the urgent need to understand and address the factors influencing adolescent mental health, particularly as the determinants of health shift in response to societal, economic, and technological changes.

**Table 2:** – WHO/UNICEF resources to support health policy development and service implementation.

Resource	Full reference
Comprehensive Mental Health Action Plan 2013–2030	Comprehensive mental health action plan 2013–2030. Geneva: World Health Organization; 2021.
The Child and Adolescent (CAMH) module in the mhGAP Intervention Guide 2.0	World Health Organization. "mhGAP intervention guide for mental, neurological and substance use disorders in non-specialized health settings: mental health Gap Action Programme (mhGAP)." mhGAP intervention guide for mental, neurological and substance use disorders in non-specialized health settings: mental health Gap Action Programme (mhGAP). 2016.
Mental health of children and young people: service guidance (WHO/UNICEF)	Mental health of children and young people: service guidance. Geneva: World Health Organization and the United Nations Children's Fund (UNICEF), 2024.
WHO Guide for integration of perinatal mental health in maternal and child health services	Guide for integration of perinatal mental health in maternal and child health services. Geneva: World Health Organization; 2022.
Helping Adolescents Thrive initiative (WHO/UNICEF)	Guidelines on mental health promotive and preventive interventions for adolescents: helping adolescents thrive. Geneva: World Health Organization; 2020.
Guidelines on mental health promotive and preventative interventions for adolescents	
HAT Toolkit	Helping adolescents thrive toolkit: strategies to promote and protect adolescent mental health and reduce self-harm and other risk behaviours. Geneva: World Health Organization and the United Nations Children's Fund (UNICEF), 2021.
Early Adolescent Skills for Emotions (EASE): group psychological help for young adolescents affected by distress in communities exposed to adversity.	Early Adolescent Skills for Emotions (EASE): group psychological help for young adolescents affected by distress in communities exposed to adversity. Geneva: World Health Organization and the United Nations Children's
UNICEF Global multisectoral operational framework	United Nations Children's Fund. Global Multisectoral Operational Framework for Mental Health and Psychosocial Support of Children, Adolescents and Caregivers Across Settings. New York, NY: UNICEF; 2022.
Community-based mental health and psychosocial support in humanitarian settings	United Nations Children's Fund. Operational guidelines on community based mental health and psychosocial support in humanitarian settings: Three-tiered support for children and families (field test version). New York, UNICEF, 2018.
Learning Brief on Mental Health and Psychosocial Support (MHPSS) in Education	United Nations Children's Fund. Learning Brief on Mental Health and Psychosocial Support (MHPSS) in Education. New York, UNICEF, 2022.
Operational Guidelines on Community-based Mental Health and Psychosocial Support in Humanitarian Settings: Three-tiered support for children and families (field test version)	United Nations Children's Fund. Operational guidelines on community based mental health and psychosocial support in humanitarian settings: Three-tiered support for children and families (field test version). New York, UNICEF, 2018.
Technical Note: Gender in Adolescent Mental Health	United Nations Children's Fund. Technical Note: Gender in Adolescent Mental Health, 2024.
The State of the World's Children 2021. On My Mind: Promoting, protecting and caring for children's mental health	United Nations Children's Fund, The State of the World's Children 2021: On My Mind – Promoting, protecting and caring for children's mental health, UNICEF, New York, October 2021.
ON MY MIND How adolescents experience and perceive mental health around the world	Johns Hopkins Bloomberg School of Public Health and United Nations Children's Fund, On My Mind: How adolescents experience and perceive mental health around the world, JHU and UNICEF, Baltimore and New York, May 2022.
UNICEF Compendium of Community Based MHPSS Resources	United Nations Children's Fund. Operational guidelines on community based mental health and psychosocial support in humanitarian settings: three-tiered support for children and families (field test version). New York: UNICEF; 2020.
Youth engaged for mental health (WHO)	Youth engaged for mental health: a framework for youth participation under the WHO Pan-European Mental Health Coalition. Copenhagen: WHO Regional Office for Europe; 2023.
WHO advocacy strategy for mental health, brain health and substance use	WHO advocacy strategy for mental health, brain health and substance use. Geneva: World Health Organization; 2024.
I Support My Friends-A training for children and adolescents on how to support a friend in distress	United Nations Children's Fund, Save the Children/MHPSS Collaborative and World Health Organization, I Support My Friends – Theory and Implementation Guide, UNICEF, New York, 2021.
Mind the gap an evidence and gap map of low- and middle-income countries	United Nations Children's Fund, Mind the gap an evidence and gap map of low- and middle-income countries, UNICEF, New York, 2022.

In addition to trends seen recently, population level trends have shown a marked increase and a marked widening of the gender gap [1,8]. Although compared to boys, girls have historically reported higher levels of internalizing mental health problems and lower levels of well-being, this gap has systematically increased over the last two decades. These trends seem to be more pronounced in Western European countries, however recent evidence indicates an overall increase in psychological problems across most countries in the WHO European Region [5]. Furthermore, these patterns coincide with increasing use of mental health services for this generation and increases in psychotropic medicine [9]. These concerning trends underscore the importance of examining the multifaceted influences driving these changes.

These declines in adolescent mental health have been associated, among others, with increases over time in parental emotional problems, youth weight-control behaviours and eating disorders, school-related stress, as well as a rise in family poverty and social inequality in the 21st Century [7]. For example, the societal pressures experienced by adolescents today are increasing and are credited to be partially responsible for this decline in adolescent mental health. The increasing emphasis on academic achievement, driven by competitive educational systems and societal expectations, places undue strain on young people [10]. This stress, which has increased over time, disproportionately affects girls [11], who often report higher levels of perfectionism and fear of failure compared to boys [8]. These effects seem to be stronger in more wealthy and gender equal countries in the WHO European Region [12].

A commonly cited factor in the decline of adolescent mental health over the last two decades is the digital transition. This has prompted major changes in societal behaviours, and a sharp rise of social media use over the past decades which has been linked to reduced real-life interactions and increased negative social comparisons [13]. While this decline in adolescent mental health began in most Western European countries around 2010 and coincides with social media's expansion, this does not confirm a causal link. Recent reviews on the impact of digital media on child mental health pointed out that robust evidence using causally informative designs is limited, and it is likely that associations will vary according to the nature of young people's interactions with their digital environments, as well as by young people's developmental maturity and sex [7]. Similarly, when looking at population levels changes, an increase over time in country level internet use from 2002 to 2018 correlates with higher burden of psychological complaints in adolescents [8], but it remains unclear whether this is causal or influenced by factors like rising individualism, perfectionism, and competitiveness. The broader societal impacts of increased internet use also remain largely unknown.

There is scientific research suggesting that more awareness and preventive efforts, reduced stigma and a greater willingness to disclose mental health issues could also inflate the presentation of increasing trends over time in mental health problems [14]. However, there is compelling evidence suggesting that the increase in mental health problems is not necessarily due to a greater willingness to report mental health problems [15].

**Fig. 2:**
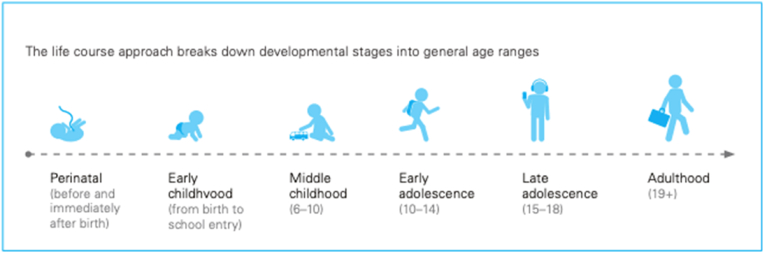
The life course approach breaks down developmental stages into general age ranges

Most importantly, this crisis is affecting critical life stages concurrently across generations (Fig. 2), both today's children and adolescents, their education and their potential, and young adults of tomorrow. This has implications for the work industry and country economies, and as such the sustainability of society, thus justifying large investments in children and young people [16]. This is of particular concern for high income countries with ageing populations who will rely on today's young people for future prosperity. Therefore, we can conclude that the declining trends in adolescent mental health over the last two decades reflect a convergence of digital, social, cultural and contextual factors. Addressing these challenges requires nuanced approaches that account for gender differences, cross-national variations, avoiding overgeneralization, and prioritizing robust, interdisciplinary research and implementation methodologies.

### 1.2 Rising demand and health system challenges

These worsening epidemiological trends in adolescent mental health have prompted an escalation in demand of health care, putting additional pressure on national health systems already stretched by many other crises, including the recent COVID-19 pandemic, the increasing cost of provision of care, and the health workforce crisis [17,18]. In addition, the widespread occurrence of new conditions - such as societal withdrawal, somatic symptoms disorder, digital addiction and others - as well as earlier onset of mental health conditions combined with uncertainties on the best treatment options [1,3] combined with higher demands due to increased use of specialized mental health care [9] exposes a gap in competencies among health professionals. This creates significant pressure on an already limited workforce in terms of numbers and competencies to address the increased patient load including both general practitioners who need to appropriately identify and refer cases, and health professional working in specialized services (paediatrics and mental health services), as well as health managers [19,20]. Health professionals working with today's children and adolescents need to respond to health conditions, often complex and chronic, for which they have not been adequately trained, and for which health services are, to a large extent, lacking trained human resources and are not appropriately organised (e.g., in multidisciplinary teams) [19,21]. Most health systems are overburdened and ill-equipped to motivate and retain health care professionals, thus exacerbating the human resources crisis [18].

Research is a pre-requisite for a well-trained and competent workforce delivering high quality of care, however only a fraction of research funding is allocated to child and adolescent mental health compared to adult projects [21]. For example, new conditions, such as societal withdrawal, require knowledge gaps to be filled by research [22,23] in identifying optimal clinical management for health professionals supporting affected children and adolescents. Equally, quality of care for paediatric mental health services more generally, needs further research, and user-informed standardised approaches [21].

Besides adding strain to the healthcare system, unmet mental health needs also place additional pressure on families, who are already stretched thin by rising living costs. In contexts where public services are lacking, patients with mental health conditions and their families may risk high out-of-pocket and catastrophic health expenses to ensure care [3,24]. Moreover, persisting social stigma and low awareness on existing effective and feasible strategies to protect and further promote mental health, limit access to care, thus creating the conditions for worst health outcomes [3].

All these aspects require a reflection to rewrite health and research priorities, and a more general call for preventive intersectoral life course strategies to improve mental health of children and adolescents. Despite investments in maternal care, investments in child and adolescent health have been reduced in many countries in recent years to accommodate other needs. More focus and cross-sector investment are required that are embedded in the life course approach from early childhood, and even better, from antenatal care, to prevent the intergenerational transmission of poor mental health (such as the increased risk of depression in children of mothers with depression), and to create effective and sustainable preventive and therapeutic models of care [25]. Investments are needed both to strengthen health services, increase workforce numbers and capacities to implement existing recommendations, and to fill existing knowledge gaps.

### 1.3 Focus on School Health Services

Schools play a crucial role in supporting adolescent mental health [26] under frameworks such as the Health Promoting Schools approach, School Health Services (SHS), and primary health care for children and adolescents [27]. To enhance mental health support in schools, efforts should focus on raising awareness, increasing knowledge and skills, and promoting destigmatization, acceptance, and help-seeking behaviours [4]. Additionally, as also outlined in Helping Adolescents Thrive [28] fostering positive interpersonal relationships in classrooms, minimizing risky behaviours, and implementing early detection strategies—both with and without formal screening—are essential. Schools should also work towards retaining students with mental health challenges to reduce absenteeism and take urgent action in cases of self-harm, aggression, or substance use incidents within school settings [26].

Comprehensive SHS should be strengthened to address adolescent mental health holistically, integrating concerns such as substance use, self-harm, and violence alongside broader health and developmental needs [29]. SHS should facilitate early identification through mental health screenings and HEADSSS (i.e., Home, Education/Employment, Eating, Activities, Drugs, Sexuality, Suicide/Depression, and Safety) assessments while offering evidence-based psychological interventions such as cognitive-behavioural therapy, problem-solving approaches, or motivational interviewing [30]. Furthermore, clinical assessments should guide referrals and support for various conditions, including behavioural disorders, anxiety and depressive disorders, eating disorders, stress, suicide risk, psychotic disorders, and substance use. Protecting student confidentiality and preventing stigma or discrimination are fundamental to ensuring that young people feel safe accessing mental health services in educational settings [26].

### 1.4 Policy recommendations

Changes in the digital, social, culture and context of adolescent life and the associated increase in demand for mental health services, against a backdrop of overburdened and under resourced health services, requires an intersectoral government response for treatment and prevention.

Governments must prioritize the mental health of children, adolescents, and young adults, recognising it as a fundamental aspect of public health [31]. A greater focus on mental health in the mission to achieve universal health coverage is critical to ensure equitable access to these services for all children and adolescents [4]. This requires a notable investment of public resources allocated to support mental health services across prevention, treatment, and promotion. Policies should define a minimum package of mental health services, specifically outlining core services that should be universally available, with budgets and responsibilities clearly allocated across relevant sectors. This entails developing national policies for child and adolescent mental health services, and the development of standards for high-quality care, aligned with international standards which are currently under way in the WHO European Region. All this work should be underpinned by a co-creation process with young people themselves [32].

Building a well-trained, supported, and adequately resourced mental health support workforce is crucial for the success of policy to improve mental health [33]. A workforce with sufficient capacity, capability and resilience is needed to meet rising demands. Clarity on roles across sectors is needed, with specific emphasis on developing mental health provision in schools. The Help Adolescents Thrive (HAT) guidelines [28] from the WHO and UNICEF highlight priority interventions for universal (all adolescents), selective (adolescents at specific risk), and prevention (adolescents with already evident early signs or symptoms of mental disorders). These guidelines are a helpful resource for mental health professionals across all sectors working to support child and adolescent mental health.

Moreover, addressing mental health conditions requires a ‘mental health in all policies’ (MHiAP) approach aimed at thoroughly understanding the different determinants of mental health through an intersectoral lens [34]. Besides intersectionality, it is key to adopt a multisectoral approach involving coordination across various sectors such as health, education, social services, and justice. For example, cross sector approaches to addressing knife crime, recognising mental health as a determinant, are needed. Poverty and poor parental mental health is a toxic combination for child mental health and subsequent knife crime or contact with the police [35,36]. Collaboration between health, education and justice at national and local level, for example through violence reduction units, is needed to develop coherent approaches. Furthermore, taking a life course intergenerational approach is needed recognising that parental mental health is a key determinant of child mental health [25]. Therefore, population level policies addressing the wider determinants of mental health are needed with a focus on welfare benefits unemployment insurance, warm housing interventions, neighbourhood renewal, paid parental leave, gender equality policies, community-based parenting programmes, and less restrictive migration policies [37]. Cross-sectoral collaboration on mental health through national and local committees with clear performance indicators [38] and sufficient and effective resource allocation is required to foster coordination and guide the implementation of policies at all levels of government [39].

A recent UNICEF report argues that protecting children's mental health in the digital age requires coordinated action by governments, the technology industry, schools, and families [40]. More specifically, preventing children from going online is not an effective solution; instead, policy must focus on reducing clear risks such as online sexual abuse and bullying, which show the strongest associations with poor mental health. Technology companies must adopt a zero-tolerance approach, investing in safety-by-design, robust moderation, and continuous child rights due diligence. Governments should strengthen legislation to hold platforms accountable for harmful or illegal content, ensuring compliance with national laws. In parallel, schools should deliver age-appropriate sexuality and online safety education, equipping children with skills to recognize and resist abuse. Training professionals across education, health, and justice sectors is also essential. Parents play a complementary role by monitoring younger children's digital use, maintaining open dialogue, and relying on trusted guidance to help older children navigate risks responsibly [40].

Several key concepts must be considered when designing policies for mental health support for young people. One is the emphasis on *early identification of mental health issues*, since early intervention can significantly reduce the risk of long-term mental health problems and improve the overall quality of life for children and adolescents [41,42]. Alongside early detection, *mental health services must be made accessible and affordable, with minimal bureaucratic barriers to care*. For example, age-appropriate help ought to be available to adolescents without the need to involve their parents [27]. Firstly, by eliminating unnecessary obstacles, young people are more likely to seek support when needed. Secondly, *a holistic and continuous approach to mental health is needed*, where not only healthcare services are involved, but also strong support systems throughout daily life [43]. Schools, cultural organizations, youth groups, and sports clubs all play a vital role in this sense by providing essential social support, reducing isolation, and fostering resilience [44]. For this reason *policies should encourage the integration of these sectors into the broader mental health framework* to ensure that young people have multiple avenues for support. Similarly, mental health aid ought to also be provided to children's caregivers, as their mental health is known to be a factor influencing youth well-being [45].

Finally, *increasing awareness of mental health issues and promoting mental health literacy* [46] *is essential for reducing stigma and encouraging help-seeking behaviour*. Policies should promote active involvement of children, adolescents, and their families in the development and delivery of mental health programs, while focusing on recovery. This participation not only improves the relevance and effectiveness of these programs but also empowers communities to take ownership of mental health care. Governments are invited to follow WHO Quality Standards for Child and Youth Mental Health Services when formulating a holistic, accessible and timely approach for support and preservation of well-being in youth. By implementing these comprehensive policies, governments can create a more supportive environment for young people's mental health, ensuring they have the care and resources needed to thrive now and in the future.

## Funding

AC was supported by the project “Research of Excellence on Digital Technologies and Wellbeing CZ.02.01.01/00/22_008/0004583” which is co-financed by the 10.13039/501100000780European Union.

## Declaration of competing interest

The authors declare that they have no known competing financial interests or personal relationships that could have appeared to influence the work reported in this paper.
